# Towards a standardised approach for evaluating guidelines and guidance documents on palliative sedation: study protocol

**DOI:** 10.1186/1472-684X-13-34

**Published:** 2014-07-07

**Authors:** Ebun Abarshi, Judith Rietjens, Augusto Caraceni, Sheila Payne, Luc Deliens, Lieve Van Den Block

**Affiliations:** 1 International Observatory on End-of-Life Care, Lancaster University, Lancaster LA1 4YG, UK; 2 Department of Public Health, Erasmus Medical Centre, Rotterdam, The Netherlands; 3 End-of-Life Care Research Group, Vrije Universiteit Brussel & Ghent University, Vrije Universiteit Brussel (VUB), Brussels, Belgium; 4 Fondazione IRCCS Istituto Nazionale dei Tumori, Milan, Italy; 5 European Palliative Care Research Center, Trondheim Norway, EAPC Research Network, Milan, Italy; 6 Vrije University Medical Center, EMGO Institute for Health and Care Research, Amsterdam, The Netherlands; 7 Department of Family Medicine, Vrije Universiteit Brussel (VUB), Brussels, Belgium

**Keywords:** Palliative sedation, Practice guidelines, Content analysis, Comparative research, Study protocol

## Abstract

**Background:**

Sedation in palliative care has received growing attention in recent years; and so have guidelines, position statements, and related literature that provide recommendations for its practice. Yet little is known collectively about the content, scope and methodological quality of these materials.

According to research, there are large variations in palliative sedation practice, depending on the definition and methodology used. However, a standardised approach to comparing and contrasting related documents, across countries, associations and governmental bodies is lacking. This paper reports on a protocol designed to enable thorough and systematic comparison of guidelines and guidance documents on palliative sedation.

**Methods and design:**

A multidisciplinary and international group of palliative care researchers, identified themes and clinical issues on palliative sedation based on expert consultations and evidence drawn from the EAPC (European Association of Palliative Care) framework for palliative sedation and AGREE II (Appraisal Guideline Research and Evaluation) instrument for guideline assessment. The most relevant themes were selected and built into a comprehensive checklist. This was tested on people working closely with practitioners and patients, for user-friendliness and comprehensibility, and modified where necessary. Next, a systematic search was conducted for guidelines in English, Dutch, Flemish, or Italian. The search was performed in multiple databases (PubMed, CancerLit, CNAHL, Cochrane Library, NHS Evidence and Google Scholar), and via other Internet resources. Hereafter, the final version of the checklist will be used to extract data from selected literature, and the same will be compiled, entered into SPSS, cleaned and analysed systematically for publication.

**Discussion:**

We have together developed a comprehensive checklist in a scientifically rigorous manner to allow standardised and systematic comparison. The protocol is applicable to all guidelines on palliative sedation, and the approach will contribute to rigorous and systematic comparison of international guidelines on any challenging topic such as this. Results from the study will provide valuable insights into common core elements and differences between the selected guidelines, and the extent to which recommendations are derived from, or match those in the EAPC framework. The outcomes of the study will be disseminated via peer-reviewed journals and directly to appropriate audiences.

## Background

In recent years, guidelines and related literature that inform the practice of intermittent or continuous sedation until the time of death in palliative care have been on the increase. However, a systematic comparison is yet to be done. The World Health Organization (WHO) advocates adequate relief from suffering as an essential part of care for the dying [1], for which reason palliative sedation could be permissible under specified conditions [2,3]. For a small but significant patient population, its use until death may be considered appropriate and therapeutic [4-6], though raising some questions about its justification and the differences in practice [7-11]. According to European studies, the frequency of deep and continuous sedation of patients until death varies somewhat, though some of such studies were conducted at different times with non-identical definitions. For instance, an incidence of 2.5% of all deaths in 2001 – 2002 (Denmark) and 16.5% of all deaths in 2007 – 2008 (UK) were recently reported [4,6].

To address this, evidence-based guidelines, protocols, frameworks, position statements and related literature have been developed, somewhat demonstrating the commitment within the care community at identifying appropriate clinical practices and at the same time, supporting practitioners [2]. However, these materials (hereafter referred to as guidance documents) vary somewhat, depending on prioritized goals [12-14], governmental [15,16], institutional [12] and societal policies [17-19]. A closer look at such documents is likely to reveal certain unique qualities and important variations.

Aside nomenclature issues [20-22], it is well understood that palliative sedation can be an important and necessary therapy, if administered prudently [2]. This practice, in some context is known as palliative sedation therapy [3,11], has been defined as "the monitored use of medications intended to induce a state of decreased or absent awareness or unconsciousness in order to relieve the burden of otherwise intractable suffering in a manner that is ethically acceptable to the patient, family and health-care providers" [2]. There appears to be a growing trend in this practice in Europe [4-6,23-27], and in the various care settings where people die [25,26,28]. Amidst relatively large country variations in prevalence, the actual practice of palliative sedation by both specialists and generalists has been widely reported [4-11,19,20,27,29]. This underscores the need to understand better regulation, policy and practice in this area. Moreover research suggests that the development and dissemination of practice guidelines do not necessarily translate into improved patient outcomes [30-32]. Likewise, methodological quality may or may not be linked to recommendation validity in practice guidelines [30,33] or overall impact of a report.

Therefore, the main objective of our study is to examine the characteristics and contents of guidelines and related literature on palliative sedation. A second objective is to explore if and how the implementation, impact and dissemination of the guidelines were evaluated. While we aim to study a wide range of guidelines from different European countries, we acknowledge that this may be a challenging task, because guidelines are seldom produced for a scientific audience or as peer-review papers. Rather, they are produced with the end-users in mind. Accessing such literature that has not been formally published (also called grey literature) can be problematic despite the fact that some of these documents, produced by government and non-government institutions, are increasingly published on the internet [34], and can therefore be retrieved via search engines i.e. Google and Google Scholar. Previous reviews had focused on more general aspects of palliative sedation [28,29,35,36], or approached the issue of recommendation from a different angle [3,37]. Studies using the AGREE II appraisal tool [33,38] focused solely on "guidelines", thus excluding literature with valuable recommendations and other potential benefits.

In this protocol, we present a standardised approach for comparing guidelines and related literature that provide care criteria and recommendations for the practice of palliative sedation; describing in detail the trajectories involved, strengths, weaknesses, and opportunities.

## Methods

This standardised method is developed for comparing guidelines and guidance documents (including protocols, frameworks, position statements and related literature that offer clinical guidance) on palliative sedation. It comprises five stages: i) identification of validated tools such as the Appraisal Guideline Research and Evaluation (AGREE II) instrument [33], and relevant themes/concepts (e.g. recommendations from the EAPC Framework on palliative sedation [2]), ii) development of a comprehensive checklist, iii) systematic collection of published and non-published or grey literature, iv) pilot testing the checklist and data extraction, and v) data analyses.

I. Stage One: identification of tools

The authors of this protocol selected themes which they considered most relevant to the practice of palliative sedation, given its multiple definitions and uses [21,22]. In order to incorporate as many variations of "sedation" practices as possible, a working definition was employed for palliative sedation: "the deliberate lowering of a patient’s consciousness with the use of sedative medication" [39]. Using the European Association of Palliative Care (EAPC) framework which addresses the eight key palliative care domains drawn up by the World Health Organization (WHO) [1] and the National Consensus Project (NCP) in USA [40], and which offers evidence-based recommendations on key clinical and ethical issues [2] they sought appropriate tools to address the objectives of the study.

The inclusion criteria included guidelines and guidance documents on sedation practices within the context of palliative care, which have been authorised for use in clinical practice.

A guideline was defined as a systematically developed statement that could be used to facilitate decision making in clinical settings [33], while a guidance document was a less formal and less structured material that simply contained recommendations. The exclusion criteria applied are as follows:

a) Language: A guideline (or guidance document) not produced in English, Dutch, Flemish or Italian. The choice of language is based on the first language of members of the research group.

b) Purpose of sedation: If the sedation practice reported in the guideline (or guidance document) does not take place within the context of palliative care delivery, i.e. if it is described as being used for overcoming noxious procedures, for burn care, and end-of-life weaning from ventilator support [2]. All relevant literature covering sedation practices performed on comparable end-of-life patient populations admitted in the ICU (intensive care unit) will be included.

c) Content: A guideline (or guidance document) that did not describe any care criteria or provide any recommendation.

d) Coverage: A guideline (or guidance document) that is not developed for a nation or region, e.g. an institutional guideline.

II. Stage Two: development of a comprehensive checklist

The checklist was developed via a multi-level process of searching literature, identifying domains, selecting instruments, ordering questions, testing and refining. The authors sought an overview of the content and recognisable domains, appropriate for assessing care in the terminal phase of life. A conceptual framework was developed to cover aspects of the actual practice of palliative sedation considered important. They identified a priori, other themes and domains that could be measured or compared [40] and consensus was reached on the aspects most relevant to the research objectives by discussion.

Existing instruments that could be applied for this purpose were sought, and where no known instrument was found, the most-appropriate questions were developed to explore the research objectives. Also they built on previous guideline development research [33]. The compiled checklist was then widely discussed, with attention paid to length, language, clarity, and the ordering of the questions. To ensure a standardised collection process, detailed instructions were developed to accompany the checklist.

### Checklist

The first and general section explores general characteristics of the selected guidance documents, followed by terms, definitions and types of palliative sedation (see Appendix). Questions on the definition of "refractory", "palliative sedation", and such terms are asked in an open-ended response fashion, such that the responses can be further researched. The second section focuses on recommendations proferred in the selected documents. For this, items from the EAPC framework for palliative sedation were adapted into questions, exploring the comparability of each document with the former’s ten-item recommendations [2]. The first part of this section comprises of yes/no categories, to assess in a general sense whether the listed recommendations are explicitly or implicitly contained in the documents, and this format was thought to be applicable to the different regions and countries likely to be represented in the study. The second part is however more detailed, exploring whether specific items have been explicitly included in the documents assessed. The third section evaluates the methodological rigour of how each guideline was developed using the AGREE II tool. The latter comprises 23 items organized in six domains: scope and purpose, stakeholder involvement, rigor of development, clarity of presentation, applicability, editorial independence; an overall quality score, and whether the guideline would be recommended for use in practice [41]. Being a generic instrument, our questions will be adapted to suit our use for appraising selected guidelines on palliative sedation, in ways suggested by its authors [33]. Then the items will be rated on a 7-point Likert scale from 1 (Strongly Disagree) which means there is no information on the specified item, to 7 (Strongly Agree) which means the full criteria has been met. A quality score will be calculated for the separate domains, and the domain score will be calculated by summing up the scores per domain and scaling their total as a percentage of the maximum possible score for that specific domain [33]. The fourth section probes the implementation and dissemination plans of the guidelines, reviews and other selected documents based on their content and a relevant literature review.

### Appraisers

Four appraisers (EA, JR, AC, LVB) will independently evaluate selected documents, written in English, Dutch, Flemish and Italian. This team of appraisers include methodologists, health scientists, and clinicians familiar with the subject of palliative sedation and guidelines. Where possible, each document will be evaluated by at least two appraisers.

III. Stage Three: selection of relevant documents

To incorporate a range of relevant published, and not-formally-published literature (also called grey literature), increasingly on the internet [34] we chose to conduct searches in multiple databases (PubMed, CancerLit, CNAHL, Cochrane Library, NHS Evidence), via search engines such as Google and Google Scholar, and involve professionals who were most likely to have come across these guidelines or relevant literature.

a) Literature search

A search in PubMed will be performed for published outputs from 1990 through May 2013 using a modified search string, originally developed by one of the authors [9] (see below). Following consultation with other authors, the search strategy was modified so as to incorporate other MeSH terms: "recommendation", "guidance", "framework", joined together by the Boolean operators (AND, OR). The title and abstract of all the search hits will be screened by the first author for relevance and based on the exclusion criteria. When unsure, a second opinion will be sought from JR or AC. The search will be modified for use in the other listed databases.

("palliative sedation" OR "terminal sedation" OR "continuous deep sedation" OR "continuous sedation") AND ("end of life" OR palliat* OR terminal* OR death OR dying*) AND ("guideline"[Publication Type] OR "guidelines as topic"[MeSH Terms] OR "guideline"[All Fields] OR "practice guideline"[Publication Type] OR "practice guidelines as topic"[MeSH Terms] OR "practice guideline"[All Fields]).

b) Web search

With the assistance of a Subject Librarian, an exploratory search will be performed in Google and Google Scholar, seeking additional guidelines, reviews and documents, that might have been missed in the above steps, or that perhaps were not published in scientific databases. To ensure the intentional inclusion of literature published in English, as well as Dutch, Flemish and Italian, we will apply the terms "palliative sedation", "terminal sedation", "dying", "end of life", "guidelines", and "framework", and the following translated terms in varying combinations:

palliatieve sedatie, richtlijn; sédation en médecine palliative, la sédation pour détresse en phase terminale ou en fin de vie, recommandations; guia de sedacion paliativa; raccomandazioni, sedazione terminale, sedazione palliative; therapeutische palliative sedierung, einsatz sedierender, leitlinie.

c) Online survey among EAPC members

A 90-day online survey designed to solicit guidelines on palliative sedation and related literature from registered EAPC national associations’ contacts (representing approx. 76000 members).

d) Manual search for references from the guidelines, reviews, and documents that emerge from the above steps (a + b + c).

Bibliographies of the articles retrieved from the above-listed steps, will be hand-searched for guidelines, review materials etc., mentioned or referred to.

IV. Stage Four: testing the checklist and data extraction

### Pilot test

The draft checklist will be tested/scored by all project group members using one of the guidelines as a sample. Final revisions will be made and we will agree on a single scoring pattern. Thereafter, all guidelines in English will be independently read and scored by the first two authors (EA and JR), and colleagues who speak the languages and are familiar with palliative care issues will assist with scoring papers in other selected languages. Scores will be sent to EA. In the unlikely case of disagreement, consensus will be reached by consulting a third reviewer AC, or if the need arises, the entire group. After this, the data derived from the checklists will be sorted and collated for data entry.

V. Stage Five: data analyses

The first author, EA will enter the data using the SPSS software with an appropriate coding and organising scheme. Data will be cleaned using SPSS syntax operations, and then analysed along the lines of the research objectives. Two researchers EA and JR will then read independently and apply content labels to identified themes/sub-themes. Labels will be compared and consensus will be reached by discussion. This process will be continued until all labels related to the research objectives have been addressed.

∎ Generation of sub-sets of comparable data will be analysed systematically: for instance.

∎ General characteristics (differences and similarities between the selected guidelines).

∎ Analyses of the open-ended question (definition of palliative sedation) and the use of terms might in a way support the debate on the need for an agreed "definition" for palliative sedation.

∎ Comparison of recommendations in selected guidelines based on the EAPC framework.

∎ Assessment of quality/comparison of quality of selected guidelines using the AGREE II instrument.

∎ Comparison of implementation and dissemination plans.

∎ Examination of related documents for other interesting features.

## Discussion

To the best of our knowledge, this is the first study protocol for a systematic comparison of guidelines and guidance documents on palliative sedation. Our methodology is thorough, systematic, and goes beyond AGREE II– i.e. it is able to capture valuable information from such literature that do not classify as "guidelines" which the AGREE II instrument is typically designed to appraise [33,38]. Strech and Schildmann demonstrated in their paper exploring the ethical quality of guidelines on palliative care, that existing tools are lacking for evaluating non-clinical aspects of guidelines and comparing salient domains (i.e. ethical content) [42]. This protocol presents a framework, which includes domains and issues that are key in the development of guidelines, providing recommendations for the care of a terminally ill patient in the last phase of life in general, but for the practice of (anticipatory) palliative sedation in particular.

### Strengths

This method has the potential to provide an objective evaluation of clinical guidelines and guidance documents on palliative sedation, and to facilitate a systematic comparison between/within countries and associations. Essentially it can provide insights into the core common components of guidance documents and their main differences. In addition, it can provide useful information about the different aspects of such guidelines and the domains covered: with elaborated details on questions, much more than the existing tools can elicit, especially if the goal is to compare contents more thoroughly. This means it can give further insight into the extent to which certain aspects of care are covered by different guidelines. Furthermore, this exercise will offer a general framework and a comprehensive instrument, which could initiate the development of palliative sedation guidelines for associations/countries that are yet to develop or adopt one. It may even foster the process of developing an European guideline for palliative sedation in the future. Lastly, it will provide insights into the extent to which recommendations in the EAPC framework are being used.

### Weaknesses

Our search for guidelines and guidance documents has a number of shortcomings (internet survey, limited searches etc.), especially because these documents often exist as grey literature. These are informally published written materials (such as reports) that are often difficult to trace via conventional methods, unlike published articles and monographs. They are nonetheless important sources of information for research, particularly because they tend to be original. Moreover, palliative care is organised differently in European countries, for a variety of reasons (financial, cultural, historical etc.); hence the different documents are likely to have slightly different interests and areas of emphases. In addition, guidelines and guidance documents are developed and produced differently, and with different foci. It is possible that the guidelines/guidance documents produced by scientific or research groups will tend to be more robust in terms of evidence, recommendations, methodological quality and dissemination. A methodological limitation is that valuable input may be missed from the papers that were not produced in the included languages (i.e. French, German, Spanish and Swedish). Also, given that the selected papers will not have been produced at the same time, it is likely that those papers produced after the EAPC framework was published (2009) will tend to achieve higher scores in the related section than those produced before; however keeping the main characteristics of the study design constant will ensure comparability of data to earlier or later framework. It will be left to see how the methodological quality combines with other characteristics of the clinical recommendations.

### Opportunities for future research

The checklist will provide the opportunity for future studies to build on the data obtained in this study and accurately identify developments in this area of palliative sedation practice. People can see common features in all guidelines and guidance documents, or use parts of the documents that are specific to their country. Perhaps members of the National Palliative Care Associations could be recruited in subsequent attempts at collecting national guidelines in the future. The contacts of many of such associations can be found in the current edition of the EAPC Atlas [43] or on the EAPC website [44]. Some guidelines are maintained in collective databases, e.g. National Institute for Health and Clinical Excellence (NICE) [45] in the UK; the CBO [46] in the Netherlands; the German Agency for Quality in Medicine (AZQ) [47]; the Belgian Pallialine initiative [48], the National Guideline Clearing House [49], and the Guidelines International Network (G-I-N) [50]. Perhaps search strategies could be designed to facilitate the extraction of data from these individual databases. Lastly, the outcome of the study will be published in peer-reviewed journals and disseminated directly to appropriate audiences.

### Opportunities for practice and policy

The outcome of the approach could guide practitioners and policy makers in making more informed choices in the implementation of a practice guideline. Also, it could provide the platform to share best practices across settings and countries.

## Conclusion

This study protocol acknowledges that guidelines can be central to the prudent and correct usage of palliative or therapeutic sedation in the management of unbearable symptoms and refractory suffering, experienced by patients already facing death. The approach proposed is a generic one that emphasizes thorough assessment of both clinical and non-clinical aspects of a guideline, which can likewise be used to evaluate other palliative care practice guidelines. It examines in detail the content of a guideline and lays a foundation for systematic evaluation and comparison. Also, it offers an opportunity for critical analysis of policies, providing recommendations that will ultimately ensure that guidelines are adequately formulated, robustly developed, and appropriately used.

## Appendix

99-item CHECKLIST on Palliative Sedation Guidelines.

## Competing interests

The authors declare that they have no competing interests.

## Authors’ contributions

EA, AC and LVDB designed the paper. EA, JR, AC, SP, LD and LVDB developed the checklist and the paper further. EA did the literature search, and led the writing of the script. All authors revised and approved the final version of the paper.

## Pre-publication history

The pre-publication history for this paper can be accessed here:

http://www.biomedcentral.com/1472-684X/13/34/prepub
